# Metagenomic Analysis of Nitrate-Reducing Bacteria in the Oral Cavity: Implications for Nitric Oxide Homeostasis

**DOI:** 10.1371/journal.pone.0088645

**Published:** 2014-03-26

**Authors:** Embriette R. Hyde, Fernando Andrade, Zalman Vaksman, Kavitha Parthasarathy, Hong Jiang, Deepa K. Parthasarathy, Ashley C. Torregrossa, Gena Tribble, Heidi B. Kaplan, Joseph F. Petrosino, Nathan S. Bryan

**Affiliations:** 1 Integrative Molecular and Biomedical Sciences Graduate Program, Baylor College of Medicine, Houston, Texas, United States of America; 2 Alkek Center for Metagenomics and Microbiome Research, Baylor College of Medicine, Houston, Texas, United States of America; 3 Department of Microbiology and Molecular Genetics, Medical School, The University of Texas Health Science Center Houston, Houston, Texas, United States of America; 4 Department of Periodontics, School of Dentistry, The University of Texas Health Science Center Houston, Houston, Texas, United States of America; 5 Department of Molecular Virology and Microbiology, Baylor College of Medicine, Houston, Texas, United States of America; 6 Texas Therapeutics Institute, Brown Foundation Institute of Molecular Medicine, The University of Texas Health Science Center Houston, Houston, Texas, United States of America; Albany Medical College, United States of America

## Abstract

The microbiota of the human lower intestinal tract helps maintain healthy host physiology, for example through nutrient acquisition and bile acid recycling, but specific positive contributions of the oral microbiota to host health are not well established. Nitric oxide (NO) homeostasis is crucial to mammalian physiology. The recently described entero-salivary nitrate-nitrite-nitric oxide pathway has been shown to provide bioactive NO from dietary nitrate sources. Interestingly, this pathway is dependent upon oral nitrate-reducing bacteria, since humans lack this enzyme activity. This pathway appears to represent a newly recognized symbiosis between oral nitrate-reducing bacteria and their human hosts in which the bacteria provide nitrite and nitric oxide from nitrate reduction. Here we measure the nitrate-reducing capacity of tongue-scraping samples from six healthy human volunteers, and analyze metagenomes of the bacterial communities to identify bacteria contributing to nitrate reduction. We identified 14 candidate species, seven of which were not previously believed to contribute to nitrate reduction. We cultivated isolates of four candidate species in single- and mixed-species biofilms, revealing that they have substantial nitrate- and nitrite-reduction capabilities. Colonization by specific oral bacteria may thus contribute to host NO homeostasis by providing nitrite and nitric oxide. Conversely, the lack of specific nitrate-reducing communities may disrupt the nitrate-nitrite-nitric oxide pathway and lead to a state of NO insufficiency. These findings may also provide mechanistic evidence for the oral systemic link. Our results provide a possible new therapeutic target and paradigm for NO restoration in humans by specific oral bacteria.

## Introduction

The human gastrointestinal tract represents a major habitat for bacterial colonization. The microbiota of the lower intestinal tract is widely recognized to play a symbiotic role in maintaining a healthy host physiology [1] by participating in nutrient acquisition and bile acid recycling, among other activities. In contrast, although the role of oral microbiota in disease is well studied, specific contributions to host health are not well defined. The entero-salivary nitrate-nitrite-nitric oxide pathway, which can positively affect nitric oxide (NO) homeostasis, represents a potential symbiotic relationship between oral bacteria and their human hosts [2], [3].

The gaseous free radical NO, which is endogenously produced in vascular endothelial cells, neurons and immune cells, plays a critical role in various physiological processes, including vascular homeostasis, neurotransmission, and host defense mechanisms, respectively. Continuous availability of NO is essential for cardiovascular system integrity. In the circulation, NO is an important regulator of vascular tone and blood pressure, and inhibits oxidative stress, platelet aggregation, and leukocyte adhesion [4]. NO insufficiency is strongly correlated with cardiovascular risk factors [5], is causal for endothelial dysfunction, and serves as a profound predictive factor for future atherosclerotic disease progression [6], [7], [8], [9] and cardiovascular events [10], [11]. In mammalian systems, NO is generated by NO synthases (NOS) from the amino acid L-arginine and molecular oxygen [12]. The entero-salivary nitrate-nitrite-NO pathway is a NOS-independent, and oxygen-independent, pathway to NO formation that is an important alternative pathway to produce bioactive NO, particularly during periods of hypoxia [13], [14], [15]. Dietary nitrate, obtained primarily from green leafy vegetables and beets, is rapidly absorbed from the upper gastrointestinal tract into the bloodstream, where it mixes with the nitrate formed from the oxidation of endogenous NO produced from mammalian NOS. Up to 25% of this nitrate is actively taken up by the salivary glands and concentrated up to 20-fold, reaching concentrations approaching 10 mM in the saliva [16]. Salivary nitrate is metabolized to nitrite via a two-electron reduction, a reaction that mammalian cells are unable to perform, during anaerobic respiration by nitrate reductases produced by facultative and obligate anaerobic commensal oral bacteria [15], [17]. Numerous studies have shown that nitrite produced from bacterial nitrate reduction is an important storage pool for NO in blood and tissues when NOS-mediated NO production is insufficient [14],[18],[19],[20],[21]. In various animal models and in humans, dietary nitrate supplementation has shown numerous beneficial effects, including a reduction in blood pressure, protection against ischemia-reperfusion damage, restoration of NO homeostasis with associated cardioprotection, increased vascular regeneration after chronic ischemia, and a reversal of vascular dysfunction in the elderly [22], [23]. Some of these benefits were reduced or completely prevented when the oral microbiota were abolished with an antiseptic mouthwash [22], [24] Additionally, it was recently shown that in the absence of any dietary modifications, a seven-day period of antiseptic mouthwash treatment to disrupt the oral microbiota reduced both oral and plasma nitrite levels in healthy human volunteers, and was associated with a sustained increase in both systolic and diastolic blood pressure [25]. Altogether, these studies firmly establish the role for oral nitrate-reducing bacteria in making a physiologically relevant contribution to host nitrite and thus NO levels, with measureable physiological effects.

Although a few nitrate reducing bacteria in the oral cavity have been identified [13], a full metagenomic analysis has not been performed. We analyzed nitrate reduction by bacterial communities present in tongue-scraping samples from healthy human volunteers during four days of *in vitro* growth and performed a parallel metagenomic analysis of these samples to identify specific bacteria associated with nitrate reduction. Through 16S rRNA gene pyrosequencing and whole genome shotgun (WGS) sequencing and analysis, we identified specific taxa that likely contribute to nitrate reduction. Preliminary biochemical characterization of nitrate and nitrite reduction by four candidate species indicates that complex community interactions contribute to nitrate reduction. The presence or absence of these select bacteria may be a new determinant of nitrite and NO bioavailability in humans and thus a new consideration for cardiovascular disease risk.

## Materials and Methods

### Subject population and microbiological sampling

**Figure 1:**
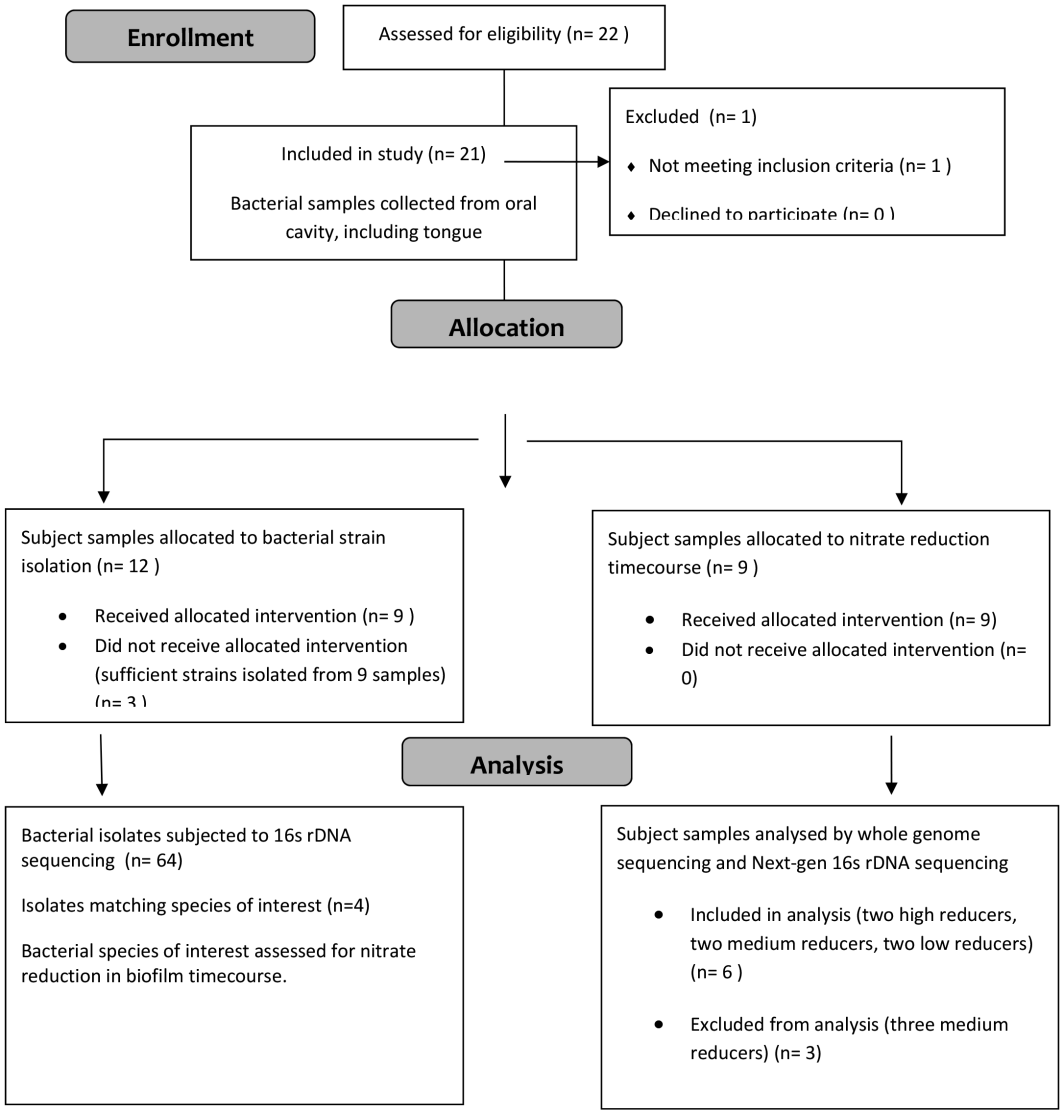
Flow diagram for study design and subject recruitment.

All human subjects research was reviewed and approved by the Committee for the Protection of Human Subjects at the University of Texas Health Science Center at Houston # HSC-DB-10-0035 & HSC-MS-12-0303. Subjects were recruited from the faculty, staff, and students of the University of Texas Health Science Center at Houston. Subjects were evaluated for oral health, including the use of a standard periodontal exam, with spot probing for bleeding and loss of attachment, and an oral health subject history. A flow chart of the study design and subject inclusion/exclusion is shown in Figure 1. Six subjects were informed of the study, signed informed consent form and enrolled, according to the following inclusion and exclusion criteria. Inclusion criteria: over the age of 18 and capable of giving consent, bleeding on probing at less than 10% of sites, greater than 24 teeth, no attachment loss of more than 4 mm, no clinical history of bone loss, no oral hard or soft tissue lesions, no use of antibiotics within the previous 3 months. Exclusion criteria: bleeding on probing at more than 10% of sites, less than 24 teeth, attachment loss of more than 4 mm at any site, clinical history of bone loss, presence of oral hard or soft tissue lesions, recent use of antibiotics within the previous 3 months. Samples were collected using a sterile stainless steel tongue scraper, passed once over the tongue dorsum from back to front with gentle pressure. Tongue-scraping samples were transferred into 1 ml of reduced transport medium (0.045% K_2_HPO_4_, 0.045% KH_2_PO_4_, 0.09% NaCl, 0.09% (NH_4_)_2_SO_4_, 0.018% MgSO_4_, 0.038% EDTA, 0.04% Na_2_CO_3_, 0.02% dithiothreitol, 0.2% Bacto-agar, 5% glycerol) in Nunc freezer vials with a sterile swab, and placed on wet ice for immediate transport to storage at −80°C. Prior to freezing, a portion of each tongue-scraping sample was dispensed into 50 µl aliquots for use as inoculum for the *in vitro* biofilm assays. An additional 50 µl aliquot was dispensed into a collection tube from the MoBio PowerSoil Kit (MoBio, Carlsbad, CA), and frozen for transport to the Human Genome Sequencing Center (HGSC) at Baylor College of Medicine (BCM) for DNA extraction and 16S rRNA gene pyrosequencing. All samples were de-identified and assigned random numbers (A73, C66, D55, E64, F76, and G77).

### In vitro biofilm assays

Aliquots of tongue-scraping samples (50 µl per well) were used as the inoculum for the generation of *in vitro* biofilm communities on sterile coated poly-methyl-methacrylate (PMMA) disks (0.4 cm) in 24-well sterile polystyrene tissue culture plates (Falcon). The PMMA disks in each well were coated with 600 ul of 20% fetal bovine serum (FBS) in carbonate buffer (pH 9.5) and incubated overnight at 4°C. The next day the FBS solution was removed and replaced with 500 ul of Biofilm Medium (BioM), composed of 45% trypticase soy broth (TSB) (Difco) supplemented with 7.6 µM hemin (Sigma) and 2.9 µM menadione (Sigma), 15% FBS, 10% phosphate buffered saline (PBS), and 12.8 mM Na_2_CO_3_. Each well was inoculated with the tongue-scrapping sample and the plate was then incubated at 37°C in an anaerobic chamber (Coy) for 24 hr, under an atmosphere of 86% N_2_, 10% CO_2_, and 4% H_2_. For nitrate-reduction assays, every 24 hr the spent medium was removed and replaced with 500 µl of fresh BioM. The recovered spent medium was centrifuged and 325 ul of the supernatant (spent medium) was transferred to a 0.5 ul tube and stored at 4°C for subsequent assessment of the nitrate and nitrite content. Spent medium was collected at 24, 48, 72, and 96 hr, and the assays were repeated independently three times. For the microbiome analysis of each sample, one PMMA disk was removed at 24, 48, 72, and 96 hr, placed directly into a MoBio PowerSoil Kit sample tube, and frozen on dry ice for transport to the CMMR at BCM.

### Assessment of nitrate and/or nitrite concentration

Each spent medium sample was added to an equal volume of ice-cold methanol, immediately vortexed and centrifuged at 13,200 rpm for 10 min to precipitate the protein and any remaining cells. Combined nitrate and nitrite (NOx) analysis was performed in the laboratory of one of the authors (N.S.B.) by a dedicated ENO-20 HPLC System (EiCom Corporation, San Diego, CA) [26]. This system is sensitive and selective for the measurement of NOx in all biological matrices and has the capacity for high throughput. In brief, to separate nitrite and nitrate, the nitrate was first reduced to nitrite through a reaction with cadmium and reduced copper inside a reduction column. The two resolved peaks were then mixed with Griess reagent (dinitrogen trioxide, N_2_O_3_, generated from acidified nitrite that reacts with sulfanilamide) in-line to form the classical diazo compound, which was then detected spectrophotometrically. Triplicate determinations were performed on each specimen and the final values were averaged.

### Microbial DNA extraction, 16S rRNA gene amplification and pyrosequencing

Bacterial genomic DNA was extracted from the initial tongue-scraping samples and the PMMA disks. DNA was extracted using the MoBio PowerSoil Kit following protocols benchmarked as part of the NIH Human Microbiome Project. The V3–V5 regions of the 16S rRNA gene were amplified using individually barcoded universal primers containing linker sequences for 454-pyrosequencing. Sequencing was performed at the HGSC at BCM using a multiplexed 454-Titanium sequencer.

### 16S data analysis

Sequence processing and analysis was performed using QIIME version 1.6.0 [27]. The sequencing file was de-multiplexed and quality filtered according to the following parameters: permitted sequence length between 200 bp and1000 bp, a required minimum average quality score of 35 over a 50 bp sliding window, no homopolymer longer than 6 bp, no ambiguous bases allowed, two primer mismatches allowed, and one barcode mismatch allowed. Quality trimming of 16S rRNA gene sequences resulted in 190,722 high quality sequences with an average of 6357 sequences per sample (for the number of sequences associated with each individual sample, refer to Table S1). Sequences were clustered *de novo* and binned into OTUs based on 97% identity (equivalent of species), assigned taxonomy using RDP Classifier trained to the GreenGenes database (October 2012 release), and singleton reads were removed from the dataset. Before alpha diversity metrics were calculated, the OTU table was subsampled to 4910 reads per sample 5 times; the average values across the 5 subsampled OTU tables were used to calculate alpha diversity metrics. Prior to beta diversity analysis, the OTU table was subsampled to 5008 reads, the smallest number of reads associated with any one sample. Unweighted UniFrac analysis was then performed to assess community similarity between samples; PCoA and Bi Plots were created from the UniFrac distance matrix to visualize sample clustering and taxa associated with clusters. ANOSIM was used to determined cluster tightness. Pie charts were used to visualize the mean relative abundances of genera present in each group of samples. Supervised machine learning using the randomForest algorithm identified specific OTUs that discriminated between groups.

### Whole genome shotgun sequencing and analysis

Based on the results of the 16S rRNA gene pyrosequencing and analysis, we chose three representative samples, one from each nitrate-reduction group, and performed whole genome shotgun (WGS) sequencing. Bacterial genomic DNA isolated from sample F76-2 (best reduction), F76-3 (intermediate reduction), and A73-4 (worst reduction) was sequenced on one lane of the Illumina HiSeq (2×150) platform at the HGSC at BCM. An average of 156.7 million reads was obtained per sample, with an average of 84.6% Q30 bases. FASTQ sequencing files were quality trimmed (leading N's removed, sequence truncated at the first N thereafter) and aligned against the human genome (hg19) and PhiX to filter out known contaminants. Using a custom perl script, the trimmed, filtered FASTQ files were interleaved into one FASTQ file, which was converted to FASTA format. To obtain taxonomic classification of the bacterial taxa present in each sample, we passed the WGS FASTA file for each sample through MetaPhlAn [28], a computational tool that relies on clade specific marker genes for taxonomic assignment of unassembled WGS data. Further, to assess gene content in these three samples, we passed the sequence data through USEARCH (32-bit version), using the KEGG v54 prokaryotic database as the reference databases, and further passed the resulting files through HUMAnN [29], a computational tool that takes BLAST/Usearch outputs and provides information about pathway coverage and abundance.

### Bacterial strain isolation, identification, and culture conditions

The strains assessed for nitrate reduction are low passage human isolates from the oral bacteria collection of GDT. The strains selected for use in this study were originally isolated from two volunteer donors, enrolled with approval from Committee for the Protection of Human Subjects at the University of Texas Health Science Center at Houston # HSC-DB-10-0035 (Collecting Oral Bacteria from Healthy Volunteers). For general strain isolation, plaque or saliva samples were serially diluted in TSB and aliquots were plated on non-selective blood agar plates. The bacterial plates were incubated at 37°C under anaerobic conditions for 48 hours to seven days. From each bacterial plate, well-isolated colonies were identified using a dissecting microscope, recovered with a sterile inoculating needle, and repeatedly sub-cultured on blood agar plates to obtain pure cultures. For each purified strain, DNA was extracted, PCR-amplified with universal 16s rDNA primers (27F, 1492R ([30]), and the resulting PCR product submitted for Sanger sequencing encompassing the V3–V5 hyper variable region (SeqWright). Sequence data was subsequently assembled, trimmed to remove low quality data, and compared to the Human Oral Microbiome Database [31] 16S rDNA RefSeq by local BLAST (CLC Genomics Workbench). Isolates were assigned to a matching genus at 95–97% identity, and a matching genus and species at >97% identity. *Veillonella dispar* UTDB 1-3 and *Fusobacterium nucleatum spp polymorphum* UTDB 1-5 were originally isolated from dental plaque from the same subject, and strains *Actinomyces odontolyticus* UTDB 59-1 and *Streptococcus mutans* UTDB 59-3 were isolated from saliva from a second subject. For this study, all four strains were grown anaerobically at 37°C in a Coy anaerobic chamber under an atmosphere of 86% N_2_, 10% CO_2_, and 4% H_2_. Routine culture medium was TSB supplemented with 5% yeast extract, 2%NaHCO_3_, 7.5 µM hemin and 3 µM menadione. TSB blood agar plates (BAP) were made with the addition of 5% sheep's blood and 1.5% agarose. The medium for *V. dispar* was supplemented with 2% lactate prior to cultivation.

### Identification of nitrate and nitrite reductase genes in the genome sequences of candidate species

We currently have the following commercially available candidate species in the laboratory: *Prevotella melaninogenica* strain D18 (ATCC 25845, GenBank Accession Number ACWY00000000.1), *Neisseria mucosa* strain C102 (ATCC 25996, GenBank Accession Number ACDX00000000.2), *Fusobacterium nucleatum* subsp. polymorphum strain F0401 (BEI HM-260D, NCBI Reference Sequence NZ_ADDB00000000.2), *Granulicatella adiacens* type strain GaD (ATCC 49175, GenBank Accession Number ACKZ00000000.1) and *Haemophilus* oral taxon 851 strain F0397 (*Haemophilus parainfluenzae*, BEI HM-469, GenBank Accession Number AGRK00000000.1). We obtained the whole genome sequences for these strains and used BLASTX to determine which of these strains encoded nitrate and/or nitrite reductase genes. We created a BLASTX reference database from the available sequences for the following nitrate and nitrite reductase genes: *nirK*, *nirB*, *nirD*, *narG*, *narL*, *narJ*, *narQ*, *narI*, *nrfF*, *nrfA*, *nrfH*, *napC*, *napB*, *napH*, *napD*, *napA*, *napG*, and *napF*. *Haemophilus* oral taxon 851 encodes both nitrate and nitrite reductase genes, while *Granulicatella adiacens*, *Prevotella melaninogenica* and *Fusobacterium nucleatum* subsp. *polymorphum* encode only nitrite reductase genes. Results were inconclusive for *Neisseria mucosa*, although it appears that it likely encodes both nitrate and nitrite reductase genes.

As we have not sequenced the isolate species described above, we could not search their genome sequences *in silico* for the presence of nitrate and nitrite reductase genes. Instead, we collected the genome sequences of all sequenced strains available on NCBI and used BLASTX, as described above, to determine which of the nitrate and nitrite reductase genes listed above were encoded by which strains. The strains we used were: *Actinomyces odontolyticus* ATCC 17982 (GenBank Accession number AAYI00000000.2), *Veillonella dispar* ATCC 17748 (GenBank Accession Number ACIK00000000.2), *Fusobacterium nucleatum* subsp. *polymorphum* F0401 (NCBI Reference Sequence NZ_ADDB00000000.2), and *Fusobacterium nucleatum* subsp. *polymorphum* ATCC 10953 (NCBI Reference Sequence NZ_AARG00000000.1). As described above, *Fusobacterium nucleatum* subsp. *polymorphum* F0401 encodes only nitrate reductase genes, and *Fusobacterium nucleatum* subsp. *polymorphum* ATCC 10953 also encodes only nitrite reductase genes. Conversely, *Actinomyces odontolyticus* ATCC 17982 encodes only nitrate reductase genes, and *Veillonella dispar* ATCC 17748 encodes both nitrate and nitrite reductase genes.

## Results

### Diversity of the human tongue microbiome from initial scrapings and biofilms grown for four days

Tongue-scraping samples from six healthy volunteers were obtained from the dorsal surface of the tongue, as it has been previously shown that most nitrate reduction occurs at this location in the oral cavity [13]. As revealed by 16S rRNA gene pyrosequencing and analysis, the tongue scrapings were diverse, with an average of 230.1 operational taxonomic units (OTUs) detected in these samples. The majority of OTUs in the original samples belonged to *Streptococcus* (20.2%+/−9.75%), *Veillonella* (14.1%+/−4.15%), *Prevotella* (11.8%+/−5.88%), *Neisseria* (10.8%+/−9.62%), and *Haemophilus* (8.64%+/−4.93%), although there was notable variation observed among the samples (Table 1). These results mirror those of the HMP Consortium's human microbiome project, which also found that *Veillonella*, *Prevotella*, *Haemophilus*, and *Streptococcus* were found in abundance on the tongue dorsum of healthy individuals [32].

**Table 1:** Top Ten Genera Present in Individual Tongue Scrapings.

Sample A73		Sample C66		Sample D55	
Genus	Abundance	Genus	Abundance	Genus	Abundance
*Haemophilus*	18.0%	*Prevotella*	21.5%	*Streptococcus*	35.7%
*Streptococcus*	15.4%	*Streptococcus*	13.1%	*Veillonella*	19.5%
*Neisseria*	15.1%	*Neisseria*	10.1%	*Prevotella*	10.4%
*Veillonella*	14.0%	*Veillonella*	8.55%	*Haemophilus*	4.55%
*Prevotella*	8.65%	*Haemophilus*	7.89%	*Actinomyces*	3.25%
*Porphyromonas*	5.19%	*Porphyromonas*	6.67%	*Leptotrichia*	2.84%
*Granulicatella*	3.04%	*Unclassified Genera (Pasturellaceae)*	5.13%	*Unclassified Genera (Gemellaceae)*	2.10%
*Unclassified Genera (Gemellaceae)*	2.38%	*[Prevotella]*	2.99%	*Granulicatella*	1.76%
*Unclassified Genera (Pasturellaceae)*	1.94%	*Leptotrichia*	2.82%	*Oribacterium*	1.46%
*Actinomyces*	1.16%	*Megasphaera*	2.21%	*Fusobacterium*	1.38%

Sample E64		Sample F76		Sample G77	
Genus	Abundance	Genus	Abundance	Genus	Abundance
*Streptococcus*	28.0%	*Neisseria*	26.9%	*Streptococcus*	18.7%
*Porphyromonas*	18.2%	*Veillonella*	11.8%	*Veillonella*	18.3%
*Veillonella*	12.2%	*Streptococcus*	10.2%	*Prevotella*	15.7%
*Haemophilus*	6.34%	*Haemophilus*	9.59%	*Neisseria*	9.82%
*Prevotella*	5.03%	*Prevotella*	9.49%	*Haemophilus*	5.47%
*Unclassified Genera (Gemellaceae)*	4.33%	*Porphyromonas*	6.62%	*Fusobacterium*	3.50%
*Neisseria*	2.69%	*Capnocytophaga*	3.38%	*Porphyromonas*	3.22%
*Fusobacterium*	2.57%	*Leptotrichia*	3.28%	*Actinomyces*	2.13%
*[Prevotella]*	1.73%	*Fusobacterium*	2.10%	*Granulicatella*	1.96%
*Capnocytophaga*	1.45%	*Actinomyces*	1.95%	*Leptotrichia*	1.69%

After the first 24 hours of biofilm incubation, an average of 82.2 OTUs were detected, equating to an average loss of 147.9 OTUs when compared to the tongue scrapings (Table 2). As the biofilms incubated over a total period of four days, a continual decrease in richness was observed, until by day four the biofilms consisted of an average of only 24.6 OTUs (Table 2). Notably, the biofilms were dominated by *Streptococcus*, in contrast with what was observed in the tongue scrapings, which are expected to represent the steady-state population in the native environment. These results suggest that streptococci are most adept at growing in this biofilm environment; however, all five of the genera that were most abundant in the original inocula (*Streptococcus*, *Veillonella*, *Prevotella*, *Neisseria*, and *Haemophilus*) were also detected in the biofilms. These data reveal that communities of bacteria change in culture (some grow and others do not) and allow us to monitor these changes and correlate changes in communities with changes in nitrate reduction in order to identify which bacteria in complex communities are primarily responsible for nitrate reduction.

**Table 2:** The number of OTUs associated with each sample at a sequencing depth of 4,910 reads is listed.

	Tongue scraping	24 hour biofilm	48 hour biofilm	72 hour biofilm	96 hour biofilm
**Subject A73**	197.6	68.4	53.6	20.8	19.4
**Subject C66**	278	128.8	88.4	58	37.6
**Subject D55**	211.2	72	35.4	33.4	16
**Subject E64**	199	62.6	52.6	33.2	24.6
**Subject F76**	247.2	92.6	132.4	69.6	32.8
**Subject G77**	247.8	71.6	55.4	31.6	17
**Average**	230.1	82.2	69.6	41.1	24.6

The OTU table was randomly subsampled to 4,910 reads per sample five times; OTU values listed are an average of the five subsamplings. Averages per time point are also listed.

### Nitrate reduction by bacterial biofilms differs between samples and decreases over time

The activity of a bacterial biofilm community can be defined based on its consumption of nutrients. We focused on nitrate metabolism; we defined biofilm nitrate reduction by the amount of nitrate remaining in the biofilm medium after 24 hours of growth. The nitrate content of the biofilm growth medium was approximately 30 µM prior to inoculation. Every 24 hours, corresponding with PMMA disc collection, the spent medium was carefully removed and replaced with fresh medium, and the amount of nitrate remaining in the spent medium was determined. It is important to note that we did not assess nitrate reduction by the initial inoculum (tongue scraping). However, the 24-hour time point is unique in that bacteria in the well include all of the bacteria from the original inoculum – those that attach to the substrate and those that do not. Thus, the nitrate consumption in the medium of the 24-hour samples represents the nitrate-reducing capacity of the entire population of the original tongue scraping - those bacteria that formed biofilms and those that did not but could still contribute to nitrate reduction at some point over the first 24 hour period. All subsequent samples (48, 72, and 96 hours) reflect the nitrate-reducing capacity of those cells either in the biofilm or previously associated with the biofilm.

**Figure 2:**
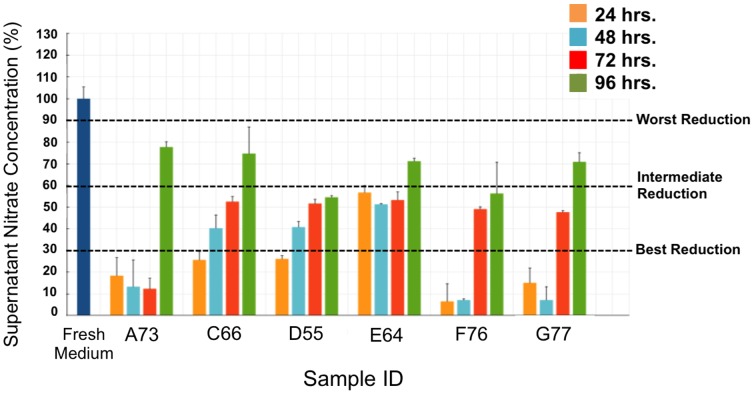
The nitrate-reducing capacity of anaerobic biofilms inoculated with tongue-scrapings samples from six healthy volunteers. Each bar represents the percentage of nitrate remaining in the spent supernatant fluid after 24; 48 hours, blue; 72 hours, orange; and 96 hours, green. The data are the average ± SEM of three individual experiments and are represented as a percent of the starting nitrate concentration in the medium. Dashed lines indicate cutoffs for placing samples in nitrate-reducing groups. Subject IDs (A73, C66, D55, E64, F76, G77) are indicated on the x-axis below each group of four bars.

There was a wide range in nitrate-reducing capacity over 96 hours across the six samples, and the longer the samples incubated, the lower the nitrate-reduction activity became, until by 96 hours only 20–45% of the activity remained (Figure 2). These changes in nitrate reductase concomitant with changes in bacterial communities in culture allowed us to investigate loss of activity with loss of specific bacteria. The samples could be separated into 4 groups based on their capacity for nitrate reduction. The first group, consisting only of sample A73, maintained a high level of nitrate reduction for the first three days. The second group, consisting of samples F76 and G77, was similar to group 1 in that these samples maintained a high level of nitrate-reducing activity for days 1 and 2, but they began to lose their activity by day 3. The third group, containing samples C66 and D55, did not efficiently reduce, even during the first 24 hours, and the little activity they had was quickly lost. The last group, containing only sample E64, never efficiently reduced nitrate. As our overall goal was to identify taxa contributing to nitrate reduction, we regrouped our biofilm samples based on their apparent nitrate-reducing activity, regardless of sample of origin or incubation time. The first group was designated “best reducers” and contained nine samples (A73-1, A73-2, A73-3, C66-1, D55-1, F76-1, F76-2, G77-1, and G77-2) that reduced at least 70% of the medium nitrate, the second group was designated “intermediate reducers” and contained ten samples (C66-2, C66-3, D55-2, D55-3, D55-4, E64-1, E64-2, E64-3, F76-3, and G77-3) that reduced between 40% and 70% of the medium nitrate, and the third group was designated “worst reducers” and contained five samples (A73-4, C66-4, E64-4, F76-4, and G77-4) that reduced less than 40% of the medium nitrate (Figure 2).

### Specific genera appear to be associated with nitrate-reduction capacity

**Figure 3:**
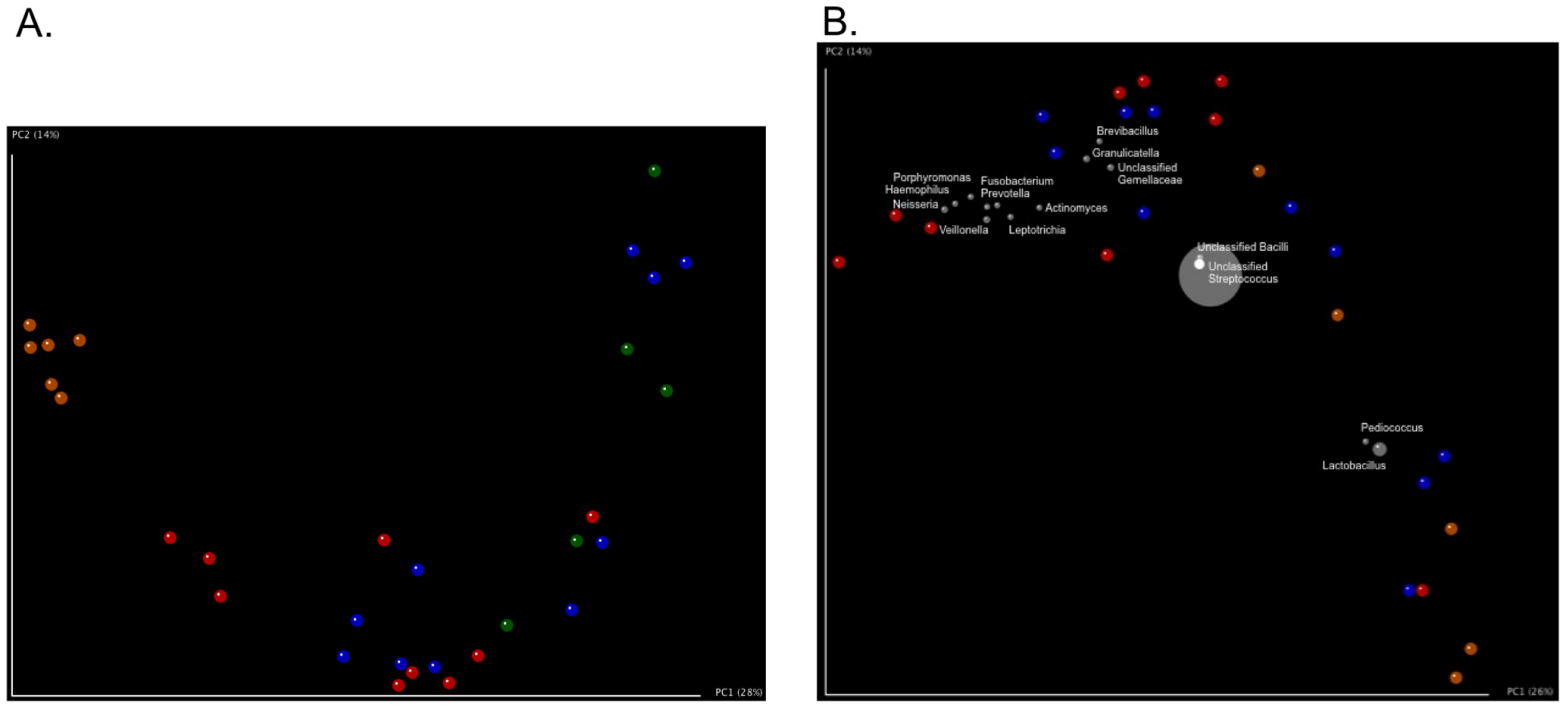
Unweighted UniFrac-based PCoA analysis reveals a gradient of samples as nitrate reduction capacity decreases with specific taxa associated with different groups. **A**) Unweighted UniFrac-based PCoA illustrates samples based on community similarity. Red dots = Best nitrate reducing samples, blue dots = intermediate nitrate reducing samples, green dots = worst nitrate reducing samples, and orange dots = inocula (original tongue scrapings). **B**) Bi Plot superimposing taxonomic information onto an unweighted UniFrac-based PCoA illustrating how similar the microbial communities of the samples are to one another. Red dots = Best nitrate-reducing samples, blue dots = intermediate nitrate-reducing samples, orange dots = worst nitrate-reducing samples, and gray dots = taxa.

To define the specific taxonomic changes in the biofilms, we first compared our samples through Unweighted UniFrac-based principal coordinates analysis (PCoA, Figure 3A). The six tongue scraping samples clustered together, indicating the initial composition of the microbial communities was similar across all subjects. However, once the tongue-scraping samples were inoculated into the biofilm environment, the community composition became more variable as the samples “fanned out” across PC1 and down PC2 without forming tight clusters based on reduction capacity (ANOSIM R statistic = 0.4701, p = 0.01). A general trend was noted in that as nitrate reduction decreased, samples moved from left to right across PC1 and up PC2. Such a gradient is not surprising, as some amount of nitrate reduction occurs in both the intermediate and worst reducing samples and thus it is likely that some of the taxa responsible for nitrate reduction in the best nitrate reducing samples are also present to some extent in the intermediate and worst nitrate reducing samples. To visualize which taxa were driving differences between samples on the PCoA plot, we generated a Bi Plot (Figure 3B). *Neisseria*, *Veillonella*, *Haemophilus*, *Porphyromonas*, *Fusobacterium*, *Prevotella*, and *Leptotrichia* were more prevalent in an area of the PCoA plot near a cluster of best nitrate-reducing samples, *Brevibacillus*, *Granulicatella*, and Unclassified *Gemellaceae* were more prevalent near a cluster consisting of best and intermediate reducing samples, and *Lactobacillus* was more prevalent in an area of the PCoA plot near worst -reducers. The presence of *Neisseria*, *Veillonella*, *Haemophilus*, *Porphyromonas*, *Fusobacterium*, *Prevotella*, *Leptotrichia*, *Brevibacillus*, and *Granulicatella* near the best nitrate reducing samples suggested that members of these genera may significantly contribute to nitrate reduction in the oral cavity.

**Figure 4:**
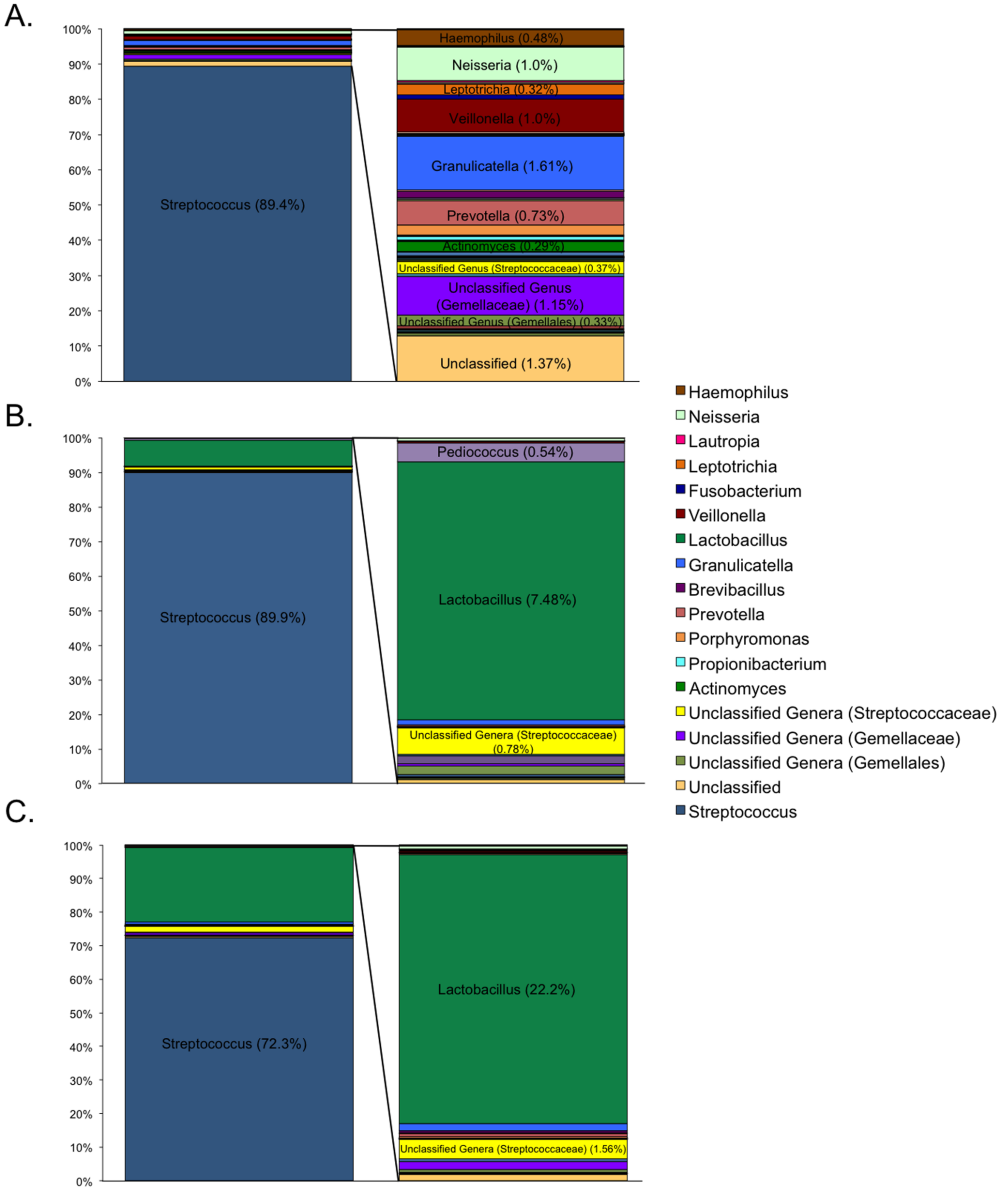
The mean relative abundance of genera present in each group of nitrate reducers. Bar charts with insets depict the mean relative abundance of genera present **A**) in the best (n = 9), **B**) intermediate (n = 10), and **C**) worst (n = 5) nitrate-reduction groups. Inset bars depict all genera detected in each group except *Streptococcus*, which was the most abundant genus detected in all groups and is depicted in the main bars. The percent abundance and taxonomic classification of the most abundance taxa are noted on the graphs.

We next examined the mean relative abundances of taxa classified to the genus level present in each nitrate-reducing group (Figure 4). The streptococci were the most abundant taxa present in all three groups, and the mean relative abundance of this taxon didn't notably change across the three groups. However, a number of taxa decreased as nitrate reduction decreased. The most notable decreases were observed in *Granulicatella* (1.61% relative abundance in the best nitrate-reducing group vs. 0.62% relative abundance in the worst nitrate-reducing group), *Veillonella* (1.0% vs. 0.15%), *Neisseria* (1.0% vs. 0.22%), *Actinomyces* (0.29% vs. 0.007%), *Prevotella* (0.73% vs. 0.26%), *Haemophilus* (0.48% vs. 0.08%), *Fusobacterium* (0.13% vs. 0.0075%), and Unclassified genera of *the* Gemellaceae family (1.15% vs. 0.64%). All of these taxa, with the exception of *Granulicatella*, clustered closely with best nitrate-reducing samples on the Bi Plot (*Granulicatella* was closer to a mixed cluster of best and intermediate reduction samples). Although *Veillonella*, *Actinomyces*, *Granulicatella*, and *Haemophilus* have been implicated in oral nitrate reduction [13], the others have not. Interestingly, although *Lactobacillus* was almost undetected in the best nitrate-reducing group (0.008%), it comprised 7.48% of the biofilm community in the intermediate nitrate-reducing group and was the second most abundant genus (22.2%) in the worst nitrate-reduction group. Notably, half of the intermediate reducing samples originated from subjects C66 and D55, which never reduced nitrate well over four days of biofilm incubation, contained large proportions of *Lactobacillus*. Thus, we speculate that *Lactobacillus* may play an inhibitory role by producing some metabolic bi-product that shuts down nitrate reduction in the community. Further studies are needed to confirm this hypothesis, as it is possible that the samples containing high levels of *Lactobacillus* may simply have a low genetic capacity for denitrification.

Our long term goal is to examine the possible clinically relevant association between nitrate-reducing bacteria and cardiac health; therefore, we used supervised machine learning to identify OTUs that discriminate between the groups with the best and worst nitrate reduction, and thus may potentially be utilized in future diagnostics. We identified 10 OTUs classified to the family or genus level that discriminated between the best and worst nitrate-reducing groups (Table S1). Importantly, 8 of the 9 best nitrate-reducing samples were classified correctly by randomForest as best nitrate-reducing samples, corresponding to an estimated classification error rate of 11.1%, and all five of the worst nitrate-reduction samples were classified correctly, corresponding to an estimated error rate of 0%. Therefore, we are confident that the randomForest-identified discriminatory OTUs are truly discriminatory and not spurious identifications. Correlating with the results discussed above, the ten discriminatory OTUs belonged to the Streptococcaceae and Gemellaceae families and the *Streptococcus*, *Haemophilus*, *Brevibacillus*, *Granulicatella*, and *Actinomyces* genera.

### Identification of species present in a subset of samples through whole genome shotgun (WGS) sequencing

To identify the species belonging to the candidate genera identified through 16S rRNA gene pyrosequencing, we performed whole genome shotgun (WGS) sequencing on a subset of samples. The DNA from one sample from each nitrate-reduction group (best, intermediate, and worst) was sequenced and the data were analyzed using MetaPhlAn, a computational tool that assigns taxonomy down to the species level and determines percent abundance based on clade-specific marker genes [28]. Comparing the 16S and WGS data for these three samples, we noted that at the phylum level, we obtained nearly the same results, detecting slightly more Proteobacteria and Actinobacteria with WGS sequencing (data not shown), and not unexpectedly, more unclassified taxa through 16S sequencing. At the genus level, we detected the same top seven genera in the 16S and WGS best and intermediate nitrate-reducing samples, albeit at slightly different relative abundances between sequencing method; conversely, most of the top ten genera detected via 16S sequencing in the worst nitrate-reducing sample were unclassified, whereas all of the top ten genera detected in this sample through WGS sequencing were assigned taxonomic classification. These small differences are likely due to the greater depth of sequencing provided by WGS sequencing, which surveys all genes rather than focusing on just one gene and facilitates more accurate taxonomic assignment.

We identified fourteen species present at an abundance of at least 0.1% in the best nitrate-reducing sample and at the highest abundance in this sample compared to the intermediate and worst reducing sample that belonged to the genera of interest that were identified through 16S rRNA gene pyrosequencing and analysis: *Granulicatella adiacens*, *Haemophilus parainfluenzae*, *Actinomyces odontolyticus*, *Actinomyces viscosus*, *Actinomyces oris*, *Neisseria flavescens*, *Neisseria mucosa*, *Neisseria sicca*, *Neisseria subflava*, *Prevotella melaninogenica*, *Prevotella salivae*, *Veillonella dispar*, *Veillonella parvula*, and *Veillonella atypica*. Additionally, *Fusobacterium nucleatum* and *Brevibacillus brevis* were designated as species of interest even though they were not at a relative abundance of at least 0.1% in the WGS best nitrate-reducing sample. Table 3 lists these 14 candidate species detected at an abundance of 0.1% or greater in the best nitrate-reducing sample, along with the abundances of each of these species in the intermediate and worst nitrate-reducing samples.

**Table 3:** The 14 candidate species detected through WGS sequencing and analysis of one representative sample from each group are listed.

	*Best nitrate reducer*	*Intermediate nitrate reducer*	*Worst nitrate reducer*
Species	% abundance	% abundance	% abundance
*Neisseria flavescens*	3.65	1.40	0.004
*Haemophilus parainfluenzae*	3.12	0.93	0.017
*Neisseria mucosa*	2.53	0.792	0.001
*Prevotella melaninogenica*	2.22	1.35	0.020
*Granulicatella adiacens*	1.56	1.16	0.941
*Veillonella dispar*	1.34	0.587	0.002
*Veillonella atypica*	0.816	0.301	0.002
*Veillonella parvula*	0.566	0.256	0.009
*Neisseria sicca*	0.369	0.146	0.0004
*Prevotella salivae*	0.189	0.071	0
*Actinomyces odontolyticus*	0.162	0.068	0.006
*Actinomyces viscosus*	0.124	0.064	0.002
*Actinomyces oris*	0.124	0.072	0.0003
*Neisseria subflava*	0.119	0.043	0

The percent abundance of the species in the all three nitrate-reducing groups is listed.

### Metabolic pathway reconstruction reveals a global uniformity in the abundances of metabolic pathways present in samples analyzed through WGS

To determine which metabolic pathways were present in the three samples analyzed through WGS, and whether any pathways were either present or absent in one sample compared to the other two, we analyzed our WGS data using MetaPhlAn, a freely available analysis tool that provides information regarding metabolic pathway coverage and abundance based on the gene content of the dataset. We observed that our three samples were very similar in terms of both pathway coverage and abundance, with only minor differences observed. The same top eight pathways were found across all samples, and the abundances of these pathways were comparable (Table S2). Importantly, the abundance of the nitrogen-metabolism pathway, while slightly lower in the worst nitrate-reduction sample (coverage was also slightly lower in this sample), did not differ drastically between the three samples (Table S2). This is in contrast to the *in vitro* data for these three samples, which showed that these samples differed notably in their ability to reduce nitrate.

### Biochemical characterization of nitrate and nitrite reduction by four species identified through WGS analysis

**Figure 5:**
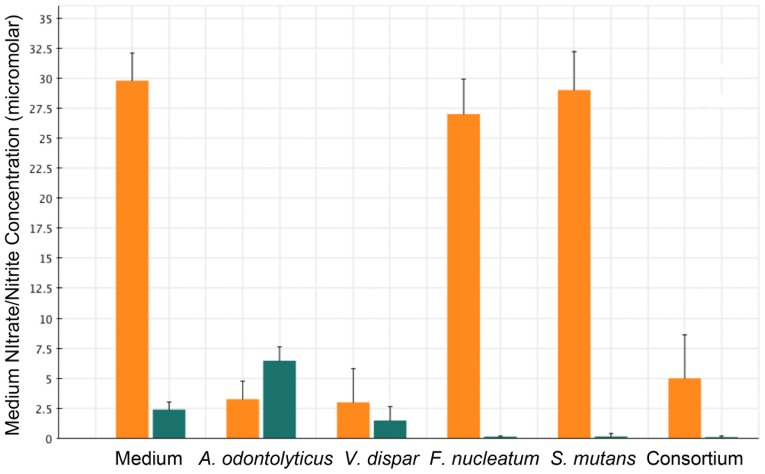
The nitrate- and nitrite-reducing capacity of four candidate species grown individually and as a consortium. Each bar represents the concentration of nitrate and nitrite remaining in the spent medium after 24(*A. odontolyticus*, *V. dispar*, *F. nucleatum*, and *S. mutans*) or a consortium of all four species at 24 hours after biofilm inoculation. The nitrate concentration, orange; nitrite concentration, green. The data are the average ± SEM of three individual experiments.

To begin to assess the nitrate and nitrite reduction by the candidate species identified through metagenomics analyses of the human tongue scrapings, we examined the *in vitro* nitrate- and nitrite-reducing capacities of recent isolates of three representative species: *Actinomyces odontolyticus*, *Fusobacterium nucleatum*, and *Veillonella dispar*, and *Streptococcus mutans* as a representative of bacteria associated with poor oral health. *A. odontolyticus* represents candidate taxa that possess only nitrate-reductase encoding genes in their genomes, *V. dispar* represents taxa that possess both nitrate- and nitrite-reductase encoding genes, and *F. nucleatum* represents taxa that possess only nitrite-reductase encoding genes. Additionally *S. mutans* also possesses only nitrite-reducing encoding genes. The strains were grown individually and as a consortium of all four strains using the same *in vitro* biofilm protocol used to grow the original tongue-scraping samples. The apparent nitrate- and nitrite-reduction activities of the species were inferred from the amount of nitrate and nitrite remaining in the spent medium (Fig. 5). As expected, both *A. odontolyticus* and *V. dispar* were effective nitrate reducers, reducing at least 80% of medium nitrate. In contrast, *S. mutans* and *F. nucleatum* did not reduce nitrate, although there were almost undetectable levels of nitrite remaining in the media of these biofilms. *V. dispar* also reduced nitrite, as expected, but not to the extent of either *S. mutans* or *F. nucleatum*. The sequenced strains of *A. odontolyticus* do not possess a nitrite-reductase gene, and in our system, nitrite levels increased in the medium of the *A. odontolyticus* biofilm compared to fresh, sterile medium, confirming that our isolated strain also does not possess a functional nitrite reductase and supporting our designation of *A. odontolyticus* as a top candidate for nitrate reduction and nitrite accumulation. The consortium of all four species exhibited good nitrate and nitrite reduction, as the nitrate levels in the spent medium were low and the nitrite levels were undetectable. However, nitrate levels were not any lower than those detected in the medium of either the *A. odontolyticus* or *V. dispar* biofilms alone, and it appeared that any nitrite released into the medium was quickly reduced by *S. mutans* and *F. nucleatum*. Therefore, creating a consortium of bacteria optimized for nitrate reduction and nitrite accumulation will require testing every candidate species individually and as consortiums to identify the inter-bacterial interactions that contribute to nitrate reduction and nitrite accumulation. These data corroborate the metagenomic indentification and the functional activity of these bacteria demonstrating that this approach can be useful for screening for specific bacteria.

## Discussion

Through 16S rRNA gene pyrosequencing and WGS sequencing and analysis, we have achieved an in-depth view of the differences between microbial biofilm communities that are good, fair, and poor at reducing nitrate. This has allowed us to identify species that likely contribute to optimal nitrate reduction in the human host that can provide the human body with continuous sources of nitrite and NO. Our findings highlight these species as potential therapeutic and/or diagnostic targets. Though others have identified specific oral nitrate-reducing bacteria [13], this is the first study to use a combination of metagenomics and biochemical techniques to gain a finer view of the oral microbiome in the context of nitrate reduction, identifying a number of taxa that were not previously implicated in oral nitrate reduction.

Previously the Doel et al., 2005 study isolated and identified five genera of oral nitrate reducing bacterial taxa on the tongues of healthy individuals: *Veillonella*, *Actinomyces*, *Rothia*, *Staphylococcus*, and *Propionibacterium* [13]. In this study, *Veillonella* species were the most abundant group of nitrate reducers isolated from the tongue, followed by *Actinomyces spp*. In our study, *Veillonella* was the most abundant nitrate-reducing genus detected in the original tongue scrapings, though *Prevotella*, *Neisseria*, and *Haemophilus* were all found at a higher abundance than *Actinomyces*, highlighting the higher resolution of our study. This difference in resolution is likely due to our use of a sequencing-based approach, which allowed us to survey the native bacterial environment on the dorsal surface of the tongue without depending on the growth requirement necessary for classic culture-based techniques. Additionally, in our study, the original samples were subsequently grown under anaerobic biofilm conditions that are more representative of the environment in the deep crypts of the tongue than the agar plates used in the Doel et al. 2005 study. A final key difference between our study and that of Doel et al. is that while Doel et al. were specifically targeting active nitrate reducers, we sought to acquire a whole-community picture, targeting all species contributing to community nitrate reduction, both directly by reducing nitrate and indirectly by acting as helper species or otherwise increasing the health and vitality of the community. Conversely we identify bacteria that may further reduce nitrite generated from nitrate reduction by species that contain nitrite reductase enzymes. Presence of these bacteria may not allow for sufficient nitrite accumulation in the saliva thereby suppressing the nitrate-nitrite-nitric oxide pathway.

An important finding of our study was not only identification of nitrate reducing bacteria but also the presence of bacterial species in these communities not genetically capable of nitrate reduction and not previously implicated in oral nitrate reduction. This poses several intriguing questions: 1) do these bacteria act as helper species, enabling nitrate reducers to more efficiently reduce nitrate? 2) do these bacteria “ride along” with nitrate reducing bacteria, feeding off a metabolic byproduct produced by nitrate reducers? and 3) do these species contribute to the health and structure of the biofilm by acting as “scaffold species” to form and maintain the biofilm? Further studies on multi-species biofilms integrating biochemical, metagenomics, and metatranscriptomics data will answer these important questions and provide more information regarding the community dynamics that contribute to oral nitrate reduction that results in nitrite accumulation.

A preliminary biochemical analysis of the nitrate- and nitrite-reduction activity of the single- and multi-species biofilms of four species identified through our metagenomic studies supports the idea that characterizing the metabolic activity of members of the community as a consortium is necessary to maximally understand and exploit their inter-bacterial interactions. In the consortium of four species, nitrate reduction did not differ from that observed by the two good nitrate reducers, indicating that at least these two species do not act additively or synergistically to reduce more nitrate than either species does alone. Whether or not other candidate species act additively or synergistically to reduce nitrate is unknown. Importantly, the two species that did not reduce nitrate well did not inhibit the ability of the two nitrate reducers in the consortium to reduce nitrate. Additionally, the amount of nitrite in the medium of the consortium was undetectable likely due to the subsequent reduction of nitrite by those bacteria that possess a nitrite reductase gene. In contrast, the optimal community would reduce the maximum amount of nitrate, while also allowing nitrite accumulation, such as was observed in the *Actinomyces odontolyticus* biofilm, to maximize the amount of bioactive nitrite available in the saliva of the host. Current theory suggests that although some members of the oral microbiome reduce nitrite, it is a slow reaction and is not generally accounted for, as the rate of nitrate reduction is fast and coupled to rapid extrusion of nitrite [13]. In contrast to this view, in our multi-species biofilm conditions, no nitrite was detected in the spent medium; however, this was a closed system, while the oral cavity is an open system in which nitrite has the means to be carried away from nitrite-reducing bacteria. Altogether, our results highlight the need to carefully characterize all of the members of a nitrate-nitrite-reducing community if we plan to optimize and maximize nitrate reduction and nitrite accumulation.

Metagenomic data is important and informative in that one can determine what a bacterial community is *capable* of doing, yet it is limited in the sense that it cannot inform what the community is *actually* doing, which can vary under different circumstances. Indeed, we observed relatively uniform bacterial communities in terms of pathway abundance across the three samples that underwent WGS analysis; however, our *in vitro* data clearly demonstrated that nitrate reduction varied widely between these samples. Thus, while each community had the same capacity for nitrate reduction, the true activity clearly differed between these communities. Our study therefore highlights that a full understanding of the entero-salivary nitrate-nitrite-NO pathway will require the generation and integration of a complete set of data from metagenomic, metatranscriptomic, metaproteomic and metametabolomic studies coupled to biochemical functional assays.

With a sample population of only six healthy individuals, we are limited in our ability to draw far-reaching conclusions; however, the preliminary results that we have obtained not only suggest that larger studies with both healthy and patient populations will add to our knowledge of nitrate-reducing bacteria and the role that they play in maintaining cardiovascular health but also provide a springboard for such studies. An outstanding key question is whether the decreased abundance or absence of nitrate-reducing communities is correlated with a state of NO insufficiency and an increased risk for cardiovascular disease. A recent study indicates that eradicating oral bacteria with anti-septic mouthwash leads to an increase in systemic blood pressure [25].There is a known correlation between oral health and systemic disease [33]. Disruption of nitrite and NO production in the oral cavity may contribute to the oral-systemic link between oral hygiene and cardiovascular risk and disease. The identification of new biomarkers for NO insufficiency and the exploitation of the oral microbiota to increase cardiovascular health will be enabled by further characterization of the enzymatic activities of native oral bacterial communities from larger healthy cohorts and specific patient populations. These cohorts should consist not only of specific U.S. population, but also of other around-the-world (European, Asian) populations. It is likely that the oral microbiomes of different ethnic groups, even those within different regions of the U.S., vary widely. It will be important to determine whether different nitrate reducing communities are more prevalent in geographically dispersed healthy populations; likewise, it will also be important to determine whether different nitrate reducing communities are lacking in specific patient populations from around the world. If certain patient populations lack specific nitrate reducing bacteria, personalized treatments to enrich for nitrate reducers may be warranted. Is it tempting to wonder whether the use of mouthwash may be discouraged as part of such treatments. Indeed, studies have shown that chlorhexadine-based bactericidal mouthwashes raise blood pressure in animal models and in humans [22], [25]; however, it has yet to be determined whether other mouthwashes, such as alcohol-based mouthwashes, have similar negative effects. Additionally, while antibiotics are sometimes used to target specific bacterial species, it is possible that potential deleterious effects of antibiotic usage on nitrate-reducing communities may preclude the use of antibiotics in specific patient populations.

Clearly, the potential for the entero-salivary nitrate-nitrite-NO pathway to serve as a NO bioavailability maintenance system by harnessing the nitrate reductase activity of specific commensal bacteria calls for studies that may be profound and truly transformative. These studies are likely to unveil new paradigms on the regulation and production of endogenous NO that are likely to be new targets for specialized, multi-faceted and potentially personalized therapeutic interventions.

## Supporting Information

Table S1 **OTUs identified through supervised machine learning (randomForest) to discriminate between best and worst nitrate reducing groups.** The OTU ID#, taxonomic classification, and mean decrease in sample classification accuracy upon removal of the OTU from the dataset are listed.
(DOCX)

Table S2 **Coverage and abundance, as determined via HUMAnN, for the top eight most abundant pathways in the three samples that underwent WGS sequencing and analysis.** Additionally, the coverage and abundance for the nitrogen metabolism pathway, which was not one of the most abundant pathways in any of the samples, is listed. The pathway coverage (presence/absence) measure is the relative confidence of each pathway being present in the sample and is expressed as a fraction between 0 and 1. The pathway abundance measure is the relative copy number of each pathway and is calculated from the gene abundance information (relative gene abundances are calculated from USEARCH results in which each read has been mapped to zero or more gene identifiers based on the quality of the match. The total weight of each read is 1.0, distributed over all gene (KO) matches by quality).
(DOCX)
