# Supplementary material for: Metagenomic Analysis of Nitrate-Reducing Bacteria in the Oral Cavity: Implications for Nitric Oxide Homeostasis
Source: PLoS One. 2014 Mar 26;9(3):e88645. doi: 10.1371/journal.pone.0088645 (PMC3966736; doi:10.1371/journal.pone.0088645)
Supplement: Table S1 — OTUs identified through supervised machine learning (randomForest) to discriminate between best and worst nitrate reducing groups. The OTU ID#, taxonomic classification, and mean decrease in sample classification accuracy upon removal of the OTU from the dataset are listed. (DOCX) [file pone.0088645.s001.docx]

Supporting Information Table 1

| **OTU ID#** | **Taxonomy** | **Mean Decrease in Accuracy (%)** |
| --- | --- | --- |
| 440 | Streptococcaceae | 1.49% |
| 759 | *Streptococcus [infantis]* | 1.19% |
| 897 | Gemellaceae | 1.05% |
| 894 | *Haemophilus [parainfluenzae]* | 1.04% |
| 589 | *Streptococcus* | 0.970% |
| 281 | Streptococcaceae | 0.888% |
| 8 | *Brevibacillus* | 0.837% |
| 1040 | *Granulicatella* | 0.809% |
| 976 | Gemellaceae | 0.804% |
| 244 | *Actinomyces* | 0.775% |

**Supplementary Table 1:** OTUs identified through supervised machine learning (randomForest) to discriminate between best and worst nitrate reducing groups. The OTU ID#, taxonomic classification, and mean decrease in sample classification accuracy upon removal of the OTU from the dataset are listed.
