# Supplementary material for: Metagenomic Analysis of Nitrate-Reducing Bacteria in the Oral Cavity: Implications for Nitric Oxide Homeostasis
Source: PLoS One. 2014 Mar 26;9(3):e88645. doi: 10.1371/journal.pone.0088645 (PMC3966736; doi:10.1371/journal.pone.0088645)
Supplement: Table S2 — Coverage and abundance, as determined via HUMAnN, for the top eight most abundant pathways in the three samples that underwent WGS sequencing and analysis. Additionally, the coverage and abundance for the nitrogen metabolism pathway, which was not one of the most abundant pathways in any of the samples, is listed. The pathway coverage (presence/absence) measure is the relative confidence of each pathway being present in the sample and is expressed as a fraction between 0 and 1. The pathway abundance measure is the relative copy number of each pathway and is calculated from the gene abundance information (relative gene abundances are calculated from USEARCH results in which each read has been mapped to zero or more gene identifiers based on the quality of the match. The total weight of each read is 1.0, distributed over all gene (KO) matches by quality). (DOCX) [file pone.0088645.s002.docx]

Supporting Information Table 2:

|  | **Best Nitrate Reducer** | | **Intermediate Nitrate Reducer** | | **Worst Nitrate Reducer** | |
| --- | --- | --- | --- | --- | --- | --- |
| Pathway | Coverage | Abundance | Coverage | Abundance | Coverage | Abundance |
| ko00471: D-Glutamine and D-glutamate metabolism | 0.833333 | 0.0321196 | 1 | 0.0323382 | 1 | 0.0323028 |
| ko00290: Valine, leucine and isoleucine biosynthesis | 0.818182 | 0.0317484 | 0.818182 | 0.032204 | 0.772727 | 0.0333221 |
| ko00550: Peptidoglycan biosynthesis | 0.72973 | 0.0267444 | 0.702703 | 0.0293467 | 0.72973 | 0.0318703 |
| ko00970: Aminoacyl-tRNA biosynthesis | 0.46875 | 0.0267403 | 0.46875 | 0.026297 | 0.453125 | 0.0262222 |
| ko03010: Ribosome | 0.381944 | 0.0267307 | 0.381944 | 0.0246808 | 0.375 | 0.0253348 |
| ko00473:  D-Alanine metabolism | 1 | 0.0252826 | 1 | 0.0268489 | 0.8 | 0.0282271 |
| ko00061:  Fatty acid biosynthesis | 0.538462 | 0.0236807 | 0.576923 | 0.0240604 | 0.538462 | 0.0260604 |
| ko00660:  C5-Branched dibasic acid metabolism | 0.583333 | 0.0227483 | 0.583333 | 0.02317 | 0.583333 | 0.0236178 |
| ko00910: Nitrogen metabolism | 0.424242 | 0.00664097 | 0.393939 | 0.0058295 | 0.30303 | 0.00523466 |

**Supplementary Table 2:** Coverage and abundance, as determined via HUMAnN, for the top eight most abundant pathways in the three samples that underwent WGS sequencing and analysis. Additionally, the coverage and abundance for the nitrogen metabolism pathway, which was not one of the most abundant pathways in any of the samples, is listed. The pathway coverage (presence/absence) measure is the relative confidence of each pathway being present in the sample and is expressed as a fraction between 0 and 1. The pathway abundance measure is the relative copy number of each pathway and is calculated from the gene abundance information (relative gene abundances are calculated from USEARCH results in which each read has been mapped to zero or more gene identifiers based on the quality of the match. The total weight of each read is 1.0, distributed over all gene (KO) matches by quality).
